# The Potential Role of Ferroptosis in Systemic Lupus Erythematosus

**DOI:** 10.3389/fimmu.2022.855622

**Published:** 2022-04-21

**Authors:** Qian Chen, Jie Wang, Mengmeng Xiang, Yilun Wang, Zhixiong Zhang, Jun Liang, Jinhua Xu

**Affiliations:** Department of Dermatology, Huashan Hospital, Fudan University, Shanghai, China

**Keywords:** systemic lupus erythematosus, ferroptosis, autoimmunity, immunity, inflammation

## Abstract

Systemic lupus erythematosus (SLE) is an autoimmune disease that is accompanied with autoantibody production and inflammation. Other features of SLE pathogenesis include iron accumulation, oxidative stress, and lipid peroxidation, which are also major biochemical characteristics of ferroptosis, a novel non-apoptotic regulated form of cell death. To date, ferroptosis has been demonstrated to be an important driver of lupus progression, and several ferroptosis inhibitors have therapeutic effect in lupus-prone mice. Given the emerging link between ferroptosis and SLE, it can be postulated that ferroptosis is an integral component in the vicious cycle of immune dysfunction, inflammation, and tissue damage in SLE pathogenesis. In this review, we summarize the potential links between ferroptosis and SLE, with the aim of elucidating the underlying pathogenic mechanism of ferroptosis in lupus, and providing a new promising therapeutic strategy for SLE.

## Introduction

Systemic lupus erythematosus (SLE), an autoimmune disease, is characterized by autoantibody production, persistent inflammation, and multiple tissue damage. This condition is induced by accumulation of cell remnants from various cell death pathways (1). Ferroptosis, a regulated necrosis process driven by iron-dependent lipid peroxidation, was first coined by Dixon et al. in 2012 (2, 3). Ferroptosis has been associated with various physiological and pathological processes, including autoimmunity [e.g., multiple sclerosis (4)], cutaneous diseases [e.g., melanoma (5, 6)] and skin wounds (7). Li et al. reported that neutrophil ferroptosis contributes to neutropenia and disease manifestations in SLE (8). The study by Li et al. is the first and only one to directly associate ferroptosis with lupus. Based on evidence from the existing limited number studies, we postulate that ferroptosis is a missing link in the vicious cycle of immune dysfunction, inflammation, and clinical manifestations in lupus. In this review, we elucidate on the significance of ferroptosis in lupus and how it may lead to inflammation and clinical manifestations.

## The Role of Iron and ROS in Ferroptosis and SLE

Ferroptosis, a non-apoptotic form of cell death, is characterized by two major biochemical characteristics: iron accumulation and lipid peroxidation (9). Iron can directly generate reactive oxygen species (ROS) through the fenton reaction or increasing the activity of iron-dependent enzymes such as lipoxygenases (LOXs) or prolyl-hydroxylases, which are responsible for synthesis of lipid peroxidation, finally leading to ferroptosis (9). This process can be suppressed by deferoxamine (DFO), an iron chelator, implying that iron-dependent ROS is the major cause of ferroptotic cell death (2).

Interestingly, it has been documented that iron metabolism and lipid peroxidation play crucial roles in autoimmunity (10, 11). Iron deposition was observed within the kidneys of lupus nephritis (LN) mice models and during human auto-inflammatory diseases (12, 13). Multiple proteins with abilities to modulate iron homeostasis have been identified to be urinary SLE biomarkers (12). The proteins mentioned above include the iron carrier proteins neutrophil gelatinase-associated lipocalin (NGAL) (14), the iron storage protein ferritin and the iron transfer protein transferrin (15). Besides, the end products of lipid peroxidation cascades are generally recognized as lipid oxidative stress biomarkers, such as malondialdehyde (MDA), 4-hydroxynonenal (HNE), conjugated dienes (CD), and isoprostanes (16). These biomarkers were found to be significantly increased and positively correlated with disease activity in SLE (17, 18), strongly implicating the important role of lipid peroxidation in immunomodulation and autoimmunity. Unregulated oxidative stress in SLE leads to immune dysfunction, abnormal cell death signals, autoantibody production, and fatal comorbidities (19, 20).

Importantly, the successful treatment of ferroptosis inhibitors in lupus-prone mice models provided direct evidence for the role of ferroptosis in lupus pathogenesis. Hepcidin, a major iron modulator and the endogenous protective molecule against ferroptosis (21), has been shown to decrease free iron availability, reduce the renal infiltration of macrophages and T cells, and further ameliorate kidney inflammation, thereby attenuating the severity of LN in lupus-prone mice models (22). Another ferroptosis inhibitor, liproxstatin-1, was shown to efficiently suppress lipid ROS levels in neutrophils and significantly attenuate lupus in mice models (8).

## Regulatory Pathways of Ferroptosis

The mechanisms and genetic networks regulating ferroptosis are complex, and are still being elucidated. The glutathione (GSH)-glutathione peroxidase 4 (GPX4) antioxidant axis is the core redox mechanism involved in ferroptosis inhibition. GSH acts as a necessary cofactor for the normal function of GPX4, an antioxidant enzyme that scavenges lipid peroxides (23). Inactivation of GPX4 by GSH depletion results in lipid peroxidation, ultimately leading to ferroptotic cell death. System $x_{c}^{-}$ (SLC7A11 and SLC3A2) is the most upstream player in the GSH/GPX4 signaling cascade. Notably, suppressed intracellular GSH and GPX levels in lupus patients are correlated with disease severity (24, 25). Reversal of GSH depletion attenuated disease severity in lupus-prone mice models (26). GPX4, a selenoprotein family member, requires selenium, a micronutrient, for its biosynthesis (27). And selenium deficiency is a risk factor for inflammation and autoimmunity, conditions that are prevalent in autoimmune diseases patients (28). GSH-GPX activity could be upregulated in lupus patients after selenium supplementation (29).

**Figure 1:**
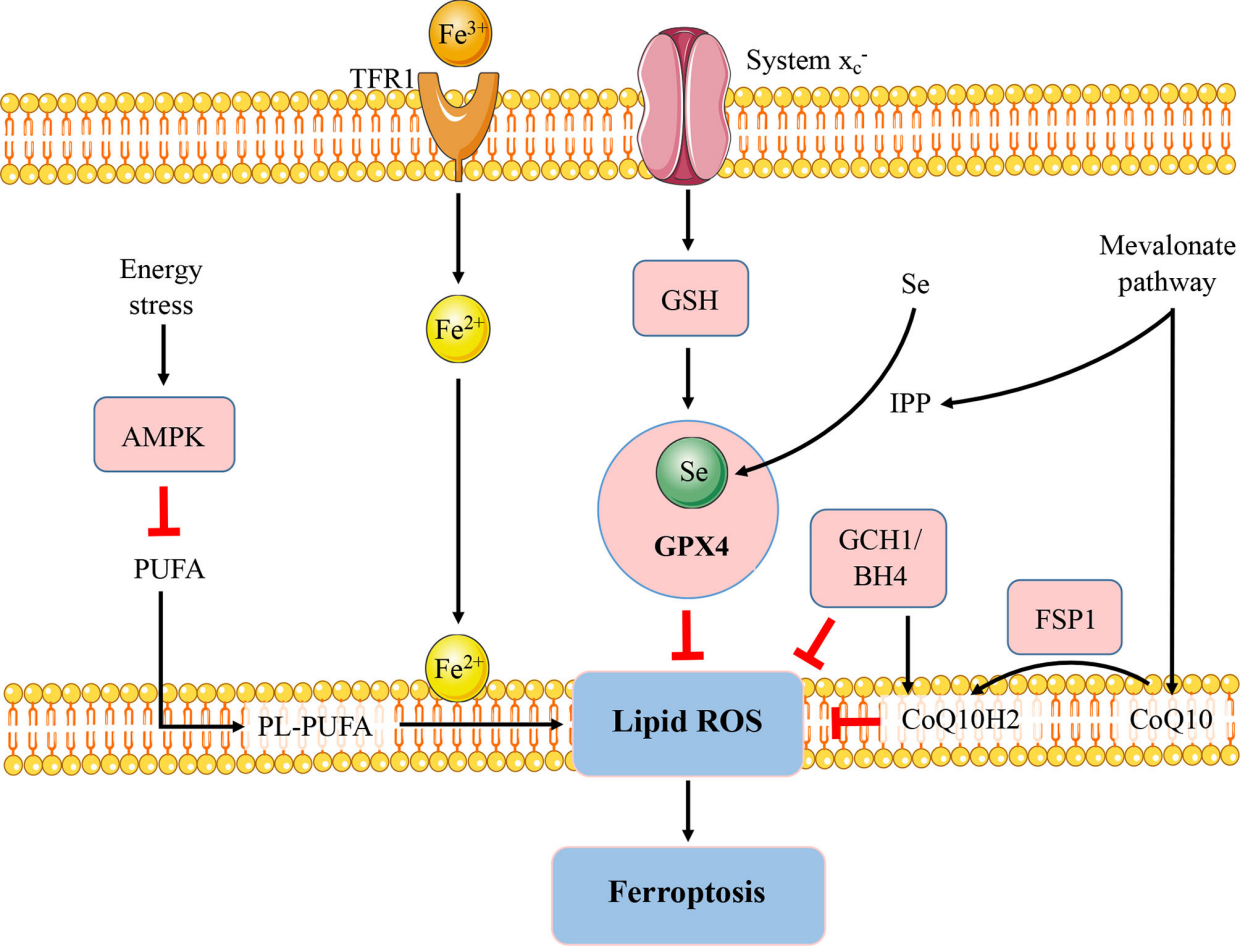
Regulatory pathways of ferroptosis. The figure briefly shows the representative pathways of ferroptosis, which are also involved in immune response and autoimmunity. GSH-GPX4, FSP1-CoQ10, and GCH1-BH4 pathways are considered as the three major stand-alone mechanisms modulating ferroptosis. The micronutrient selenium is required for biosynthesis of GPX4. CoQ10 is another important antioxidant molecule which can be reduced to CoQ10H2 by FSP1 and hence protect the cells from ferroptosis. The GCH1-BH4 axis suppresses ferroptosis by regulating the antioxidant BH4, CoQ10, and lipid peroxidation. In addition, AMPK plays an energy stress-mediated protective role against ferroptosis. Further, the mevalonate pathway can generate anti-ferroptotic biomolecules such as CoQ10 and IPP to participate in ferroptosis regulation. FSP1, ferroptosis suppressor protein 1; GCH1; GTP cyclohydrolase-1; BH4, tetrahydrobiopterin; Se, selenium; TfR, transferrin receptor; PUFA, polyunsaturated fatty acid; PL-PUFA, phospholipid containing polyunsaturated fatty acid chain. IPP, isopentenyl-pyrophosphate.

Apart from the GSH-GPX4 axis, various signaling pathways with the ability to modulate ferroptosis have been identified and associated with immune modulation and autoimmunity (**Figure 1**). AMP-activated protein kinase (AMPK), a sensor of cellular energy status, plays an energy stress-mediated protective role against ferroptosis (30), also as a key role in immune related diseases (31). AMPK activation exerts functions in metformin treatment of lupus by inhibiting B cell differentiation into germinal center and plasma cells (32). Another powerful antioxidant, coenzyme Q10 (CoQ10), which has shown beneficial effects in autoimmune diseases (33), can suppress lipid peroxidation and ferroptosis (34). The CoQ10 analog idebenone has been demonstrated that can attenuate murine lupus by modulating mitochondrial biology and reducing inflammation (35).

## The Potential Role of Ferroptosis in Lupus Immunity

Most immune cell types are implicated in SLE pathogenesis, beyond the activation of B cells (36). The significance of ferroptosis in immune systems has been reported by various studies. During maturation, activation, and differentiation of immune cells, iron metabolism and lipid peroxidation are important signaling molecules (10, 37). These processes can be regulated by antioxidant molecules such as GSH and GPX4 (38). Therefore, we discussed the relationship between ferroptosis and immunity, with a focus on SLE-associated immune cells.

T cells in lupus patients have been correlated with abnormal mitochondrial hyperpolarization and adenosine triphosphate (ATP) depletion, which cause predisposition to death by necrosis (39). Swollen lymph nodes of lupus patients harbor increased numbers of necrotic T cells, leading to inflammation and tissue damage in SLE (39–41). GSH levels are lower in T cells from patients with SLE, and the reduction degrees of GSH are associated with mitochondrial hyperpolarization and increased reactive oxygen intermediates production (42). In particular, increased intracellular iron has been found in lupus CD4+ T cells compared with healthy controls (43). Based on these findings, the possibility of ferroptosis, one of the regulated necrosis, to contribute in lupus T cells can be proposed. Besides, both CD4^+^ and CD8^+^ T cells that lack GPX4 would rapidly accumulate membrane lipid ROS, and undergo ferroptosis, leading to their inability to expand and protect against viral and parasite infections (44).

B cells are the central elements of humoral immunity and protection due to their ability to produce antibodies. Aberrant activation and differentiation of B cells with pathogenic autoantibody production are recognized as pivotal roles in the immunopathogenesis of SLE (45). Compared to hepcidin-treated lupus mice models, as previously stated, the spleens of vehicle treated group contained anomalous dense iron deposits in B-cell regions (22). Iron plays an important role in B cell maturation, germinal center formation and immune responses (46). Higher ROS levels are essential for the process of B cell activation and differentiation (37). Lipid peroxidation induced by erastin, the classical ferroptosis activator, can promote the proliferation and differentiation of human peripheral blood mononuclear cells (PBMCs) into B cells and natural killer (NK) cells (47). These findings imply that ferroptosis may govern B cell differentiation and activity through lipid peroxidation. Nevertheless, the roles of ferroptosis in B cells remain unclear. Current research demonstrated that GPX4 is indispensable for innate-like B cells rather than follicular B2 cells to prevent ferroptosis (48). Given the importance and complexity of B cells in lupus development, there is a need to establish the significance of ferroptosis in B cells.

The function of macrophages is to eliminate pathogens and maintain immune homeostasis. Activated macrophages are traditionally classified into two main subsets: the pro-inflammatory subset (classically activated macrophages, M1) and the anti-inflammatory subset (alternatively activated macrophages, M2). Monocytes from SLE patients exhibit a remarkable pro-inflammatory (M1-like) profile, which is skewed towards the anti-inflammatory (M2-like) phenotype after recovery (49). Compared to M2 macrophages, M1 macrophages express higher levels of inducible nitric oxide synthase (iNOS), leading to higher resistance to ferroptosis (50). It may explain the imbalance in macrophage polarization during lupus progression: M1 phenotypes display significant defiance against ferroptosis, yet they can survive, release proinflammatory cytokines, and fulfill their functions as “destroyers”; while M2 phenotypes are vulnerable to ferroptotic cell death induced by the loss of GPX4 activity (51).

Neutrophils are the first responders of immune defense against a broad range of pathogens (52). Currently, the research about the link between neutrophils and lupus is mainly focused on neutrophil extracellular traps (NETs), the fibrous networks protruding from activated neutrophils in response to infection or inflammation (53). However, recent study by Li et al. demonstrated that neutrophil death is majorly associated with ferroptosis in SLE, instead of NETosis, the process of NET release. Through downregulated expression of GPX4 and elevated lipid ROS levels, neutrophil ferroptosis leads to stimulation of autoreactive B cells and plasmacytoid dendritic cells (pDCs), autoantibody and type I interferon (IFN) production, finally contributing to disease manifestations (8). Therefore, ferroptosis promotes lupus progression through immune system regulation.

## The potential Role of Ferroptosis in Lupus Inflammation and Tissue Damage

**Figure 2:**
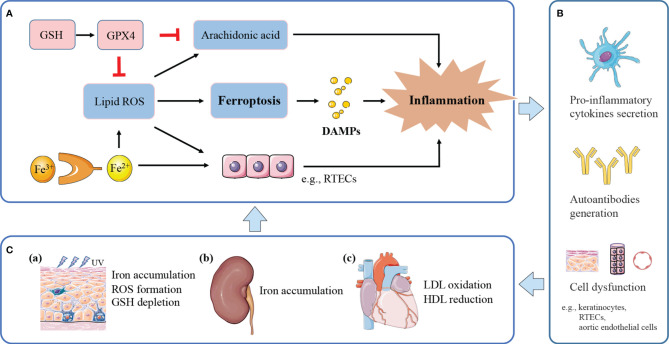
The potential model of ferroptosis in lupus inflammation and manifestations. **(A)** Ferroptosis releases DAMPs to trigger inflammation. Iron and ROS accumulation promote a pro-inflammatory environment. Massive lipid ROS released by ferroptosis helps to convert arachidonic acid to inflammatory mediators. GPX4 suppresses inflammation by inhibiting arachidonic acid oxidation and lipid peroxidation. **(B)** Ferroptotic cell death and induced inflammation exert causative effects in SLE through pro-inflammatory cytokines secretion, and autoantibodies generation, finally leading to cell dysfunction and tissue damage. **(C) **(a) Inflammation induced by UV irradiation amplifies inflammatory and immune responses, eventually causing cutaneous lesions. UVB-exposed skin lesions exhibit iron accumulation, excessive ROS and GSH depletion, leading to keratinocytes ferroptosis. (b) Persistent inflammation and immune complexes deposition accelerate lupus progression to renal failure. Kidneys uptake excessive iron in the renal tubules and undergo ferroptosis under pathological conditions. (c) Lipid peroxidation and induced inflammation contribute to endothelial dysfunction and cardiovascular injury. Lupus patients with progressive atherosclerosis show decreased HDL and increased oxLDL, which may further promote ferroptosis in aortic endothelial cells.

Ferroptosis occurs in various immune cells and affects immune response as it has been described earlier. Further, ferroptosis regulates on how immune system deals with dying cells and remnants, through the release of damage-associated molecular patterns (DAMPs) or lipid oxidation products (9). DAMPs bind to cellular receptors such as pattern recognition receptors (PRRs), upregulate stress response mechanisms, and release various cytokines and chemokines, finally leading to tissue injury and inflammation (54). For example, the signals of high-mobility group box 1 (HMGB1), one of prototypical DAMPs released by ferroptotic cells, can be integrated by advanced glycosylation end-product specific receptor (AGER) to trigger inflammation and amplify immune responses (55). HMGB1 released by ferroptosis is implicated in multiple tissue damage, including ultraviolet B (UVB)-induced keratinocyte death (56), and high glucose-exposed mesangial cell death (57). Interestingly, HMGB1 activity plays a markable role in a variety of lupus phenotypes, including LN, neuropsychiatric lupus (58), and skin lesions (59). HMGB1 exerts its causative effects in SLE through both innate and adaptive immunity (58, 60), including macrophage polarization, pro-inflammatory cytokines secretion, and autoantibodies generation. Besides, iron accumulation can directly polarize macrophages to pro-inflammatory profile (61), promote pro-inflammatory cytokine secretion to induce autoimmune diseases (13); ROS facilitates inflammatory disease *via* pro-inflammatory change (62). Massive lipid oxidative mediators released by ferroptosis directly promote the activity of cyclooxygenases (COXs) and LOXs, which convert arachidonic acid to inflammatory mediators; this process can be suppressed by GPX4 (63). Therefore, it is speculated that ferroptosis may exert its pathogenic effect in SLE by excessive inflammation, which enhances immune response, leading to organ damage and clinical manifestations. A potential model is proposed for the role of ferroptosis in lupus inflammation and induced comorbidities (**Figure 2**).

With respect to skin, keratinocyte death by ferroptosis plays a remarkable role in driving skin inflammation after UVB exposure (56). Skin lesions suffered from UVB irradiation shows elevated iron content (64), excessive accumulation of lipid peroxides, and GSH depletion, therefore undergoing ferroptosis in keratinocytes, and then leads to cutaneous necroinflammation and injury (56). Furthermore, UVB-induced skin damage can be protected by GSH and GPX4 through suppressing oxidant stress, inflammation responses, and cell death (65). Based on the lupus photosensitivity, and ROS accumulation in all cutaneous subtypes of lupus (66), the cutaneous lesions may be associated with dysregulation of iron metabolism and the consequent ferroptosis induced by UV irradiation.

LN is one of the most severe organ manifestations of lupus, which most patients would develop within 5 years of SLE diagnosis (67). Tubulointerstitial damage is recognized as one of the pathological features of the lupus kidney, and tubulointerstitial inflammation is important in the assessment and prognosis of LN (68, 69). Within this local microenvironment, renal tubular epithelial cells (RTEC) are central effector cells, driving interstitial inflammation and renal damage (70). As mentioned above, renal iron accumulation occurs in LN and contributes to the development of albuminuria (12). RTECs reabsorb the majority of filtered iron (71), and these cells have been shown to undergo ferroptosis under pathological conditions (72, 73). Treatment of lupus mice models with iron metabolism regulators, such as deferiprone and hepcidin, could mitigate kidney inflammation and delay lupus progression (12, 22). Besides, uncontrolled ROS accumulation in RTECs results in inflammation and fibrosis, leading to renal damage and chronic kidney disease progression (74). Thus, it could be speculated that iron accumulation in RTECs may exacerbate inflammatory responses by ROS formation, and synergistically accelerate progression to renal failure. Meanwhile, inflammation and oxidative stress can upregulate the expression of iron carriers and transporters, possibly causing excessive uptake of iron in the renal tubules and consequent iron-induced kidney injury (75).

For cardiovascular system, the oxidation of low-density lipoproteins (oxLDL) by ROS and the activation of endothelial cells in the artery, are recognized as initiation of atherosclerosis in SLE (76). Endothelial cells stimulated by oxLDL release inflammatory cytokines, induce chronic inflammation, finally leading to endothelial dysfunction and cardiovascular injury (76). In addition, lupus patients with progressive atherosclerosis exhibit decreased levels of high-density lipoprotein (HDL) and dysfunctional HDL (77). HDL is a natural antioxidant agent and act as a protective mechanism of atherosclerosis in SLE, protecting LDL from oxidation by ROS in the arterial intima (76, 78). Recently, a study by Bai et al. used ferroptosis inhibitor, ferrostatin-1, to treat high-fat diet-induced atherosclerosis (79). They found that Fer-1 could alleviate atherosclerosis lesion and rescue endothelial dysfunction, through inhibition of iron accumulation and lipid peroxidation, and upregulation the expression of SLC7A11 and GPX4. Compelling evidence links ferroptosis to the initiation and progression of atherosclerosis.

## Conclusion and Perspectives

In conclusion, ferroptosis is speculated to be an integral component in the vicious cycle of immune dysfunction, inflammation, and tissue damage in lupus. This review article indicates that ferroptosis has outstanding research prospects in the progression of SLE. However, it is suggested that more future studies should be conducted to fill the knowledge gaps of the relationship between ferroptosis and SLE, shed more light on the pathogenesis of SLE, as well as provide a new perspective on ferroptosis-based immunotherapy for SLE.

## Author Contributions

Conceptualization: QC, JW, JL, and JX. Funding Acquisition: JW, JL, and JX. Methodology: QC, JW, JL, MX, YW, ZZ, and JX. Supervision: QC, JW, JL, MX, YW, ZZ, and JX. Writing – Original Draft Preparation: QC. Writing - Review and Editing: QC and JW. All authors assisted with the development of the manuscript and gave final approval for publication.

## Funding

This work was supported by grants from the National Natural Science Foundation of China (grant no. 81773324, 81872526); and the Startup Foundation of Huashan Hospital (2020QD007).

## Conflict of Interest

The authors declare that the research was conducted in the absence of any commercial or financial relationships that could be construed as a potential conflict of interest.

## Publisher’s Note

All claims expressed in this article are solely those of the authors and do not necessarily represent those of their affiliated organizations, or those of the publisher, the editors and the reviewers. Any product that may be evaluated in this article, or claim that may be made by its manufacturer, is not guaranteed or endorsed by the publisher.

## Abbreviations

SLE, Systemic lupus erythematosus; ROS, reactive oxygen species; LOXs, lipoxygenases; DFO, deferoxamine; LN, lupus nephritis; NGAL, neutrophil gelatinase-associated lipocalin; MDA, malondialdehyde; HNE, hydroxynonenal; CD, conjugated dienes; GSH, glutathione; GPX4, glutathione peroxidase 4; AMPK, AMP-activated protein kinase; CoQ10, coenzyme Q10; ATP, adenosine triphosphate; PBMCs, peripheral blood mononuclear cells; NK cells, natural killer cells; iNOS, inducible nitric oxide synthase; NETs, neutrophil extracellular traps; pDCs, plasmacytoid dendritic cells, IFN, interferon, DAMPs, damage-associated molecular patterns; PRRs, pattern recognition receptors; HMGB1, high-mobility group box 1, AGER, advanced glycosylation end-product specific receptor; UV, ultraviolet; COXs, cyclooxygenases; LDL, low-density lipoprotein; RTEC, renal tubular epithelial cells; oxLDL, oxidized low-density lipoprotein; HDL, high-density lipoprotein; FSP1, ferroptosis suppressor protein 1; GCH1, GTP cyclohydrolase-1; BH4, tetrahydrobiopterin; Se, selenium; TfR, transferrin receptor; PUFA, polyunsaturated fatty acid; PL-PUFA, phospholipid containing polyunsaturated fatty acid chain; IPP, isopentenyl- pyrophosphate.

## References

[1] Mahajan A Herrmann M Muñoz LE. Clearance Deficiency and Cell Death Pathways: A Model for the Pathogenesis of SLE. Front Immunol (2016) 7:35. doi: 10.3389/fimmu.2016.00035 26904025 PMC4745266

[2] Dixon SJ Lemberg KM Lamprecht MR Skouta R Zaitsev EM Gleason CE. Ferroptosis: An Iron-Dependent Form of Nonapoptotic Cell Death. Cell (2012) 149(5):1060–72. doi: 10.1016/j.cell.2012.03.042 PMC3367386 22632970

[3] Gao M Yi J Zhu J Minikes AM Monian P Thompson CB. Role of Mitochondria in Ferroptosis. Mol Cell (2019) 73(2):354–63.e3. doi: 10.1016/j.molcel.2018.10.042 30581146 PMC6338496

[4] Hu CL Nydes M Shanley KL Morales Pantoja IE Howard TA Bizzozero OA. Reduced Expression of the Ferroptosis Inhibitor Glutathione Peroxidase-4 in Multiple Sclerosis and Experimental Autoimmune Encephalomyelitis. J Neurochem (2019) 148(3):426–39. doi: 10.1111/jnc.14604 PMC6347488 30289974

[5] Ashrafizadeh M Mohammadinejad R Tavakol S Ahmadi Z Roomiani S Katebi M. Autophagy, Anoikis, Ferroptosis, Necroptosis, and Endoplasmic Reticulum Stress: Potential Applications in Melanoma Therapy. J Cell Physiol (2019) 234(11):19471–9. doi: 10.1002/jcp.28740 31032940

[6] Gagliardi M Cotella D Santoro C Corà D Barlev NA Piacentini M. Aldo-Keto Reductases Protect Metastatic Melanoma From ER Stress-Independent Ferroptosis. Cell Death Dis (2019) 10(12):902. doi: 10.1038/s41419-019-2143-7 31780644 PMC6883066

[7] Telorack M Meyer M Ingold I Conrad M Bloch W Werner S. A Glutathione-Nrf2-Thioredoxin Cross-Talk Ensures Keratinocyte Survival and Efficient Wound Repair. PloS Genet (2016) 12(1):e1005800. doi: 10.1371/journal.pgen.1005800 26808544 PMC4726503

[8] Li P Jiang M Li K Li H Zhou Y Xiao X. Glutathione Peroxidase 4-Regulated Neutrophil Ferroptosis Induces Systemic Autoimmunity. Nat Immunol (2021) 22(9):1107–17. doi: 10.1038/s41590-021-00993-3 PMC8609402 34385713

[9] Tang D Chen X Kang R Kroemer G. Ferroptosis: Molecular Mechanisms and Health Implications. Cell Res (2021) 31(2):107–25. doi: 10.1038/s41422-020-00441-1 PMC8026611 33268902

[10] Bowlus CL. The Role of Iron in T Cell Development and Autoimmunity. Autoimmun Rev (2003) 2(2):73–8. doi: 10.1016/s1568-9972(02)00143-x 12848962

[11] Perricone C De Carolis C Perricone R. Glutathione: A Key Player in Autoimmunity. Autoimmun Rev (2009) 8(8):697–701. doi: 10.1016/j.autrev.2009.02.020 19393193

[12] Marks ES Bonnemaison ML Brusnahan SK Zhang W Fan W Garrison JC. Renal Iron Accumulation Occurs in Lupus Nephritis and Iron Chelation Delays the Onset of Albuminuria. Sci Rep (2017) 7(1):12821. doi: 10.1038/s41598-017-13029-4 28993663 PMC5634457

[13] Wang Z Yin W Zhu L Li J Yao Y Chen F. Iron Drives T Helper Cell Pathogenicity by Promoting RNA-Binding Protein PCBP1-Mediated Proinflammatory Cytokine Production. Immunity (2018) 49(1):80–92.e7. doi: 10.1016/j.immuni.2018.05.008 29958803

[14] Hinze CH Suzuki M Klein-Gitelman M Passo MH Olson J Singer NG. Neutrophil Gelatinase-Associated Lipocalin Is a Predictor of the Course of Global and Renal Childhood-Onset Systemic Lupus Erythematosus Disease Activity. Arthritis Rheum (2009) 60(9):2772–81. doi: 10.1002/art.24751 PMC3064260 19714584

[15] Conca W Al-Hakim M Moussa N Al-Salam S Corr P. Hyperferritinemia in a Woman With Systemic Lupus Erythematosus, Severe Nephritis and an Iron-Rich Intraspinal Schwannoma Mimicking Lupus Myelopathy. Clin Exp Rheumatol (2009) 27(5):834–7. 19917169

[16] Niki E. Biomarkers of Lipid Peroxidation in Clinical Material. Biochim Biophys Acta (2014) 1840(2):809–17. doi: 10.1016/j.bbagen.2013.03.020 23541987

[17] Ames PR Alves J Murat I Isenberg DA Nourooz-Zadeh J. Oxidative Stress in Systemic Lupus Erythematosus and Allied Conditions With Vascular Involvement. Rheumatol (Oxf) (1999) 38(6):529–34. doi: 10.1093/rheumatology/38.6.529 10402073

[18] Mansour RB Lassoued S Gargouri B El Gaïd A Attia H Fakhfakh F. Increased Levels of Autoantibodies Against Catalase and Superoxide Dismutase Associated With Oxidative Stress in Patients With Rheumatoid Arthritis and Systemic Lupus Erythematosus. Scand J Rheumatol (2008) 37(2):103–8. doi: 10.1080/03009740701772465 18415766

[19] Shah D Mahajan N Sah S Nath SK Paudyal B. Oxidative Stress and its Biomarkers in Systemic Lupus Erythematosus. J BioMed Sci (2014) 21(1):23. doi: 10.1186/1423-0127-21-23 24636579 PMC3995422

[20] Perl A. Oxidative Stress in the Pathology and Treatment of Systemic Lupus Erythematosus. Nat Rev Rheumatol (2013) 9(11):674–86. doi: 10.1038/nrrheum.2013.147 PMC4046645 24100461

[21] Swaminathan S. Iron Homeostasis Pathways as Therapeutic Targets in Acute Kidney Injury. Nephron (2018) 140(2):156–9. doi: 10.1159/000490808 PMC6165684 29982259

[22] Scindia Y Wlazlo E Ghias E Cechova S Loi V Leeds J. Modulation of Iron Homeostasis With Hepcidin Ameliorates Spontaneous Murine Lupus Nephritis. Kidney Int (2020) 98(1):100–15. doi: 10.1016/j.kint.2020.01.025 32444136

[23] Ursini F Maiorino M. Lipid Peroxidation and Ferroptosis: The Role of GSH and Gpx4. Free Radic Biol Med (2020) 152:175–85. doi: 10.1016/j.freeradbiomed.2020.02.027 32165281

[24] Hassan SZ Gheita TA Kenawy SA Fahim AT El-Sorougy IM Abdou MS. Oxidative Stress in Systemic Lupus Erythematosus and Rheumatoid Arthritis Patients: Relationship to Disease Manifestations and Activity. Int J Rheum Dis (2011) 14(4):325–31. doi: 10.1111/j.1756-185X.2011.01630.x 22004228

[25] Gheita TA Kenawy SA. Measurement of Malondialdehyde, Glutathione, and Glutathione Peroxidase in SLE Patients. Methods Mol Biol (2014) 1134:193–9. doi: 10.1007/978-1-4939-0326-9_14 24497363

[26] Bergamo P Maurano F Rossi M. Phase 2 Enzyme Induction by Conjugated Linoleic Acid Improves Lupus-Associated Oxidative Stress. Free Radic Biol Med (2007) 43(1):71–9. doi: 10.1016/j.freeradbiomed.2007.03.023 17561095

[27] Jiang X Stockwell BR Conrad M. Ferroptosis: Mechanisms, Biology and Role in Disease. Nat Rev Mol Cell Biol (2021) 22(4):266–82. doi: 10.1038/s41580-020-00324-8 PMC8142022 33495651

[28] Sahebari M Rezaieyazdi Z Khodashahi M. Selenium and Autoimmune Diseases: A Review Article. Curr Rheumatol Rev (2019) 15(2):123–34. doi: 10.2174/1573397114666181016112342 30324883

[29] Brown AC. Lupus Erythematosus and Nutrition: A Review of the Literature. J Ren Nutr (2000) 10(4):170–83. doi: 10.1053/jren.2000.16323 11070144

[30] Lee H Zandkarimi F Zhang Y Meena JK Kim J Zhuang L. Energy-Stress-Mediated AMPK Activation Inhibits Ferroptosis. Nat Cell Biol (2020) 22(2):225–34. doi: 10.1038/s41556-020-0461-8 PMC7008777 32029897

[31] Wang J Li Z Gao L Qi Y Zhu H Qin X. The Regulation Effect of AMPK in Immune Related Diseases. Sci China Life Sci (2018) 61(5):523–33. doi: 10.1007/s11427-017-9169-6 29127585

[32] Lee SY Moon SJ Kim EK Seo HB Yang EJ Son HJ. Metformin Suppresses Systemic Autoimmunity in Roquin(san/san) Mice Through Inhibiting B Cell Differentiation Into Plasma Cells *via* Regulation of AMPK/mTOR/Stat3. J Immunol (2017) 198(7):2661–70. doi: 10.4049/jimmunol.1403088 PMC5357783 28242651

[33] López-Pedrera C Villalba JM Patiño-Trives AM Luque-Tévar M Barbarroja N Aguirre M. Therapeutic Potential and Immunomodulatory Role of Coenzyme Q(10) and Its Analogues in Systemic Autoimmune Diseases. Antioxid (Basel) (2021) 10(4):600. doi: 10.3390/antiox10040600 PMC8069673 33924642

[34] Doll S Freitas FP Shah R Aldrovandi M da Silva MC Ingold I. FSP1 Is a Glutathione-Independent Ferroptosis Suppressor. Nature (2019) 575(7784):693–8. doi: 10.1038/s41586-019-1707-0 31634899

[35] Blanco LP Pedersen HL Wang X Lightfoot YL Seto N Carmona-Rivera C. Improved Mitochondrial Metabolism and Reduced Inflammation Following Attenuation of Murine Lupus With Coenzyme Q10 Analog Idebenone. Arthritis Rheumatol (Hoboken NJ) (2020) 72(3):454–64. doi: 10.1002/art.41128 PMC7050361 31566908

[36] Morel L. Immunometabolism in Systemic Lupus Erythematosus. Nat Rev Rheumatol (2017) 13(5):280–90. doi: 10.1038/nrrheum.2017.43 28360423

[37] Zhang H Wang L Chu Y. Reactive Oxygen Species: The Signal Regulator of B Cell. Free Radical Biol Med (2019) 142:16–22. doi: 10.1016/j.freeradbiomed.2019.06.004 31185253

[38] Mak TW Grusdat M Duncan GS Dostert C Nonnenmacher Y Cox M. Glutathione Primes T Cell Metabolism for Inflammation. Immunity (2017) 46(4):675–89. doi: 10.1016/j.immuni.2017.03.019 28423341

[39] Xu Y Shen J Ran Z. Emerging Views of Mitophagy in Immunity and Autoimmune Diseases. Autophagy (2020) 16(1):3–17. doi: 10.1080/15548627.2019.1603547 30951392 PMC6984455

[40] Kojima M Nakamura S Morishita Y Itoh H Yoshida K Ohno Y. Reactive Follicular Hyperplasia in the Lymph Node Lesions From Systemic Lupus Erythematosus Patients: A Clinicopathological and Immunohistological Study of 21 Cases. Pathol Int (2000) 50(4):304–12. doi: 10.1046/j.1440-1827.2000.01052.x 10849316

[41] Perl A Gergely P Jr. Nagy G Koncz A Banki K. Mitochondrial Hyperpolarization: A Checkpoint of T-Cell Life, Death and Autoimmunity. Trends Immunol (2004) 25(7):360–7. doi: 10.1016/j.it.2004.05.001 PMC4034110 15207503

[42] Perl A Gergely P Jr. Banki K. Mitochondrial Dysfunction in T Cells of Patients With Systemic Lupus Erythematosus. Int Rev Immunol (2004) 23(3-4):293–313. doi: 10.1080/08830180490452576 15204090

[43] Zhao M Li MY Gao XF Jia SJ Gao KQ Zhou Y. Downregulation of BDH2 Modulates Iron Homeostasis and Promotes DNA Demethylation in CD4(+) T Cells of Systemic Lupus Erythematosus. Clin Immunol (2018) 187:113–21. doi: 10.1016/j.clim.2017.11.002 29113828

[44] Matsushita M Freigang S Schneider C Conrad M Bornkamm GW Kopf M. T Cell Lipid Peroxidation Induces Ferroptosis and Prevents Immunity to Infection. J Exp Med (2015) 212(4):555–68. doi: 10.1084/jem.20140857 PMC4387287 25824823

[45] Nie Y Zhao L Zhang X. B Cell Aberrance in Lupus: The Ringleader and the Solution. Clin Rev Allergy Immunol (2021) In Press. doi: 10.1007/s12016-020-08820-7 33534064

[46] Jiang Y Li C Wu Q An P Huang L Wang J. Iron-Dependent Histone 3 Lysine 9 Demethylation Controls B Cell Proliferation and Humoral Immune Responses. Nat Commun (2019) 10(1):2935. doi: 10.1038/s41467-019-11002-5 31270335 PMC6610088

[47] Wang D Xie N Gao W Kang R Tang D. The Ferroptosis Inducer Erastin Promotes Proliferation and Differentiation in Human Peripheral Blood Mononuclear Cells. Biochem Biophys Res Commun (2018) 503(3):1689–95. doi: 10.1016/j.bbrc.2018.07.100 PMC6179365 30049441

[48] Muri J Thut H Bornkamm GW Kopf M. B1 and Marginal Zone B Cells But Not Follicular B2 Cells Require Gpx4 to Prevent Lipid Peroxidation and Ferroptosis. Cell Rep (2019) 29(9):2731–44.e4. doi: 10.1016/j.celrep.2019.10.070 31775041

[49] Wang J Xie L Wang S Lin J Liang J Xu J. Azithromycin Promotes Alternatively Activated Macrophage Phenotype in Systematic Lupus Erythematosus *via* PI3K/Akt Signaling Pathway. Cell Death Dis (2018) 9(11):1080. doi: 10.1038/s41419-018-1097-5 30348950 PMC6197274

[50] Piattini F Matsushita M Muri J Bretscher P Feng X Freigang S. Differential Sensitivity of Inflammatory Macrophages and Alternatively Activated Macrophages to Ferroptosis. Eur J Immunol (2021) 51(10):2417–29. doi: 10.1002/eji.202049114 PMC9290640 34272880

[51] Kapralov AA Yang Q Dar HH Tyurina YY Anthonymuthu TS Kim R. Redox Lipid Reprogramming Commands Susceptibility of Macrophages and Microglia to Ferroptotic Death. Nat Chem Biol (2020) 16(3):278–90. doi: 10.1038/s41589-019-0462-8 PMC7233108 32080625

[52] Liew PX Kubes P. The Neutrophil’s Role During Health and Disease. Physiol Rev (2019) 99(2):1223–48. doi: 10.1152/physrev.00012.2018 30758246

[53] Chapman EA Lyon M Simpson D Mason D Beynon RJ Moots RJ. Caught in a Trap? Proteomic Analysis of Neutrophil Extracellular Traps in Rheumatoid Arthritis and Systemic Lupus Erythematosus. Front Immunol (2019) 10:423. doi: 10.3389/fimmu.2019.00423 30915077 PMC6421309

[54] Murao A Aziz M Wang H Brenner M Wang P. Release Mechanisms of Major DAMPs. Apoptosis (2021) 26(3-4):152–62. doi: 10.1007/s10495-021-01663-3 PMC8016797 33713214

[55] Wen Q Liu J Kang R Zhou B Tang D. The Release and Activity of HMGB1 in Ferroptosis. Biochem Biophys Res Commun (2019) 510(2):278–83. doi: 10.1016/j.bbrc.2019.01.090 30686534

[56] Vats K Kruglov O Mizes A Samovich SN Amoscato AA Tyurin VA. Keratinocyte Death by Ferroptosis Initiates Skin Inflammation After UVB Exposure. Redox Biol (2021) 47:102143. doi: 10.1016/j.redox.2021.102143 34592565 PMC8487085

[57] Wu Y Zhao Y Yang HZ Wang YJ Chen Y. HMGB1 Regulates Ferroptosis Through Nrf2 Pathway in Mesangial Cells in Response to High Glucose. Biosci Rep (2021) 41(2):BSR20202924. doi: 10.1042/bsr20202924 33565572 PMC7897919

[58] Liu T Son M Diamond B. HMGB1 in Systemic Lupus Erythematosus. Front Immunol (2020) 11:1057. doi: 10.3389/fimmu.2020.01057 32536928 PMC7267015

[59] Abdulahad DA Westra J Limburg PC Kallenberg CG Bijl M. HMGB1 in Systemic Lupus Erythematosus: Its Role in Cutaneous Lesions Development. Autoimmun Rev (2010) 9(10):661–5. doi: 10.1016/j.autrev.2010.05.015 20546955

[60] Schaper F Westra J Bijl M. Recent Developments in the Role of High-Mobility Group Box 1 in Systemic Lupus Erythematosus. Mol Med (2014) 20(1):72–9. doi: 10.2119/molmed.2014.00019 PMC3960395 24531837

[61] Recalcati S Locati M Gammella E Invernizzi P Cairo G. Iron Levels in Polarized Macrophages: Regulation of Immunity and Autoimmunity. Autoimmun Rev (2012) 11(12):883–9. doi: 10.1016/j.autrev.2012.03.003 22449938

[62] Su LJ Zhang JH Gomez H Murugan R Hong X Xu D. Reactive Oxygen Species-Induced Lipid Peroxidation in Apoptosis, Autophagy, and Ferroptosis. Oxid Med Cell Longev (2019) 2019:5080843. doi: 10.1155/2019/5080843 31737171 PMC6815535

[63] Sun Y Chen P Zhai B Zhang M Xiang Y Fang J. The Emerging Role of Ferroptosis in Inflammation. BioMed Pharmacother (2020) 127:110108. doi: 10.1016/j.biopha.2020.110108 32234642

[64] Bissett DL Chatterjee R Hannon DP. Chronic Ultraviolet Radiation-Induced Increase in Skin Iron and the Photoprotective Effect of Topically Applied Iron Chelators. Photochem Photobiol (1991) 54(2):215–23. doi: 10.1111/j.1751-1097.1991.tb02009.x 1780358

[65] Shi X Shang F Zhang Y Wang R Jia Y Li K. Persimmon Oligomeric Proanthocyanidins Alleviate Ultraviolet B-Induced Skin Damage by Regulating Oxidative Stress and Inflammatory Responses. Free Radic Res (2020) 54(10):765–76. doi: 10.1080/10715762.2020.1843651 33108915

[66] Safi R Al-Hage J Abbas O Kibbi AG Nassar D. Investigating the Presence of Neutrophil Extracellular Traps in Cutaneous Lesions of Different Subtypes of Lupus Erythematosus. Exp Dermatol (2019) 28(11):1348–52. doi: 10.1111/exd.14040 31529548

[67] Anders HJ Saxena R Zhao MH Parodis I Salmon JE Mohan C. Lupus Nephritis. Nat Rev Dis Primers (2020) 6(1):7. doi: 10.1038/s41572-019-0141-9 31974366

[68] Hong S Healy H Kassianos AJ. The Emerging Role of Renal Tubular Epithelial Cells in the Immunological Pathophysiology of Lupus Nephritis. Front Immunol (2020) 11:578952. doi: 10.3389/fimmu.2020.578952 33072122 PMC7538705

[69] Gomes MF Mardones C Xipell M Blasco M Solé M Espinosa G. The Extent of Tubulointerstitial Inflammation is an Independent Predictor of Renal Survival in Lupus Nephritis. J Nephrol (2021) 34(6):1897–905. doi: 10.1007/s40620-021-01007-z 33721269

[70] Liu BC Tang TT Lv LL Lan HY. Renal Tubule Injury: A Driving Force Toward Chronic Kidney Disease. Kidney Int (2018) 93(3):568–79. doi: 10.1016/j.kint.2017.09.033 29361307

[71] Wlazlo E Mehrad B Morel L Scindia Y. Iron Metabolism: An Under Investigated Driver of Renal Pathology in Lupus Nephritis. Front Med (Lausanne) (2021) 8:643686. doi: 10.3389/fmed.2021.643686 33912577 PMC8071941

[72] Linkermann A Skouta R Himmerkus N Mulay SR Dewitz C De Zen F. Synchronized Renal Tubular Cell Death Involves Ferroptosis. Proc Natl Acad Sci USA (2014) 111(47):16836–41. doi: 10.1073/pnas.1415518111 PMC4250130 25385600

[73] Wang Y Bi R Quan F Cao Q Lin Y Yue C. Ferroptosis Involves in Renal Tubular Cell Death in Diabetic Nephropathy. Eur J Pharmacol (2020) 888:173574. doi: 10.1016/j.ejphar.2020.173574 32976829

[74] Irazabal MV Torres VE. Reactive Oxygen Species and Redox Signaling in Chronic Kidney Disease. Cells (2020) 9(6):1342. doi: 10.3390/cells9061342 PMC7349188 32481548

[75] Martines AM Masereeuw R Tjalsma H Hoenderop JG Wetzels JF Swinkels DW. Iron Metabolism in the Pathogenesis of Iron-Induced Kidney Injury. Nat Rev Nephrol (2013) 9(7):385–98. doi: 10.1038/nrneph.2013.98 23670084

[76] Skaggs BJ Hahn BH McMahon M. Accelerated Atherosclerosis in Patients With SLE–Mechanisms and Management. Nat Rev Rheumatol (2012) 8(4):214–23. doi: 10.1038/nrrheum.2012.14 PMC3765069 22331061

[77] Kim SY Yu M Morin EE Kang J Kaplan MJ Schwendeman A. High-Density Lipoprotein in Lupus: Disease Biomarkers and Potential Therapeutic Strategy. Arthritis Rheumatol (2020) 72(1):20–30. doi: 10.1002/art.41059 31350818 PMC6935404

[78] Xepapadaki E Zvintzou E Kalogeropoulou C Filou S Kypreos KE. τhe Antioxidant Function of HDL in Atherosclerosis. Angiology (2020) 71(2):112–21. doi: 10.1177/0003319719854609 31185723

[79] Bai T Li M Liu Y Qiao Z Wang Z. Inhibition of Ferroptosis Alleviates Atherosclerosis Through Attenuating Lipid Peroxidation and Endothelial Dysfunction in Mouse Aortic Endothelial Cell. Free Radic Biol Med (2020) 160:92–102. doi: 10.1016/j.freeradbiomed.2020.07.026 32768568

